# External-stimuli-assisted photocatalysis for solar chemical conversion

**DOI:** 10.1007/s44371-026-00874-4

**Published:** 2026-07-16

**Authors:** Yiyang Li, Mengqi Duan, Wentian Niu

**Affiliations:** Department of Chemistry, Wolfson Catalysis Centre, University of Oxford, Oxford, OX1 3QR UK

**Keywords:** External stimuli photocatalysis, Charge and spin dynamics, Field-catalyst interactions, Multi-stimuli catalysis, Solar-to-chemical conversion

## Abstract

Heterogeneous photocatalysis offers a promising route for solar-to-chemical energy conversion, yet its efficiency continues to be constrained by rapid electron-hole recombination and sluggish surface redox kinetics. Recent advances show that integrating external stimuli, such as electric, magnetic, thermal, microwave fields, can provide dynamically tuneable driving forces that influence charge, spin, and lattice behaviour in semiconductors. These external-stimuli interactions enable new mechanisms for promoting charge carrier separation, stabilising intermediates, and modulating interfacial energetics beyond the limits of conventional band-structure engineering. This review presents a comprehensive discussion of external-stimuli-assisted heterogeneous photocatalysis. We examine how different fields interact with semiconductors through various pathways. Representative material platforms, including metal oxides, polar-faceted supports, two-dimensional chalcogenides, are analysed to reveal structure-activity relationships. We further discuss emerging synergistic effects in multi-stimuli systems, where coupled fields create non-linear enhancements in photocatalytic performance. Advances in operando characterisation techniques are highlighted as essential tools for probing these dynamic processes. Together, these developments illustrate how external stimuli can be harnessed to design adaptive, field-responsive photocatalytic systems capable of significantly higher activity, selectivity, and stability. By outlining mechanistic principles, material design strategies, and key challenges, this review provides a framework for developing next-generation solar-to-chemical energy technologies.

## Introduction

Heterogeneous photocatalysis using semiconductor catalysts, inspired by the pioneering work of Honda and Fujishima on photoelectrochemical water splitting in 1972, has become a central research area at the intersection of chemistry, materials science, and physics [1–4]. By directly converting sunlight into chemical fuels and high-value chemicals, photocatalysis promises a sustainable approach to hydrogen evolution, CO_2_ reduction, nitrogen fixation, and pollutant degradation [5–9]. Despite decades of progress, however, the overall efficiency of photocatalytic systems remains too low for large-scale application. A key limitation arises from the intrinsic mismatch between ultrafast photophysical charge carrier dynamics and much slower interfacial redox reactions [10–12]. Photogenerated electrons and holes tend to recombine within nanoseconds before they can participate in surface catalysis, meaning that only a small fraction reaches active sites. Additional challenges include the poor utilisation of visible light – which makes up over 45% of the solar spectrum – and the sluggish kinetics of multi-electron processes such as the oxygen evolution reaction (OER) [13, 14]. Conventional approaches to address these issues have focused on material modifications, including band-gap engineering via cation or anion doping, heterojunction construction, cocatalyst deposition, and defect engineering [15–18]. While these methods can enhance performance, they are inherently static: once a material is synthesised, its internal electric field, band structure, and surface chemistry are largely fixed. To achieve more flexible control, a new strategy is needed, one that enables in-situ, tuneable, and spatially controlled manipulation of charge carriers and surface reactions.

External stimuli provide such an opportunity. When an electric, magnetic, or microwave field is applied to a photocatalytic system, it introduces additional forces that act on charge carriers, the crystal lattice, or chemical species. These effects can induce polarisation, spin alignment, or localised potential and concentration gradients [19]. Because the strength and direction of these fields can be easily tuned without altering the catalyst composition, they offer a dynamic means to promote charge separation, modify reaction pathways, and even tune product selectivity. The integration of light with these external stimuli defines the emerging concept of external stimuli-assisted heterogeneous photocatalysis. Over the past decade, research in this area has expanded rapidly [19–23]. These works established the mechanistic foundation for field-driven photocatalysis and stimulated widespread interest in exploring how electric, magnetic, and electromagnetic fields influence photocatalytic reactions. Subsequent investigations have progressed from empirical demonstrations to microscopic-level understanding. However, most studies have focused primarily on charge separation under applied fields and have evaluated field effects largely through pollutant degradation systems, leaving other important chemical and materials-level aspects less explored.

Unlike earlier reviews that centred mainly on charge dynamics, this review emphasises the chemical foundations and material design of field-responsive photocatalytic systems. Previous reviews have primarily focused on charge-separation mechanisms under electric or magnetic fields [19–23], whereas the present review adopts a broader perspective that includes thermal-assisted photocatalysis, microwave effects, field-responsive interfaces, operando characterization, and coupled-field systems. Particular attention is given to the liquid-solid and catalyst-support interfaces near the active photocatalyst. Moreover, we extend our discussion beyond conventional photocatalysis by adapting field-effect concepts from related areas such as thermal catalysis and electrocatalysis, where external stimuli have been shown to modulate reaction energetics, adsorption behaviour, and spin polarisation. Drawing these parallels will provide a broader mechanistic understanding and suggests new strategies for designing photocatalysts that can exploit similar field-induced phenomena. The combination of external stimuli with rationally engineered catalysts therefore offers a promising route to achieve finer control over photocatalytic reactivity and selectivity. This review therefore provides a comprehensive and systematic framework for understanding and optimising external stimuli effects in heterogeneous photocatalysis. Specifically, we:

- Consolidate the mechanistic principles through which external electric, magnetic, thermal, microwave stimuli influence semiconductor charge, spin, lattice, and interfacial behaviours, highlighting the common physical interactions that underpin field-induced enhancements.
- Analyse how these field effects can be strengthened through rational catalyst and reactor design, including defect engineering, polar-faceted supports, plasmonic or magnetic components, high-temperature condition, and tailored interfacial architectures.
- Compare the influence of various external stimuli on the fundamental steps of photocatalysis, photon absorption, charge-carrier generation, separation and transport, spin dynamics, and surface redox kinetics, across representative material classes.
- Review extensive case studies spanning hydrogen evolution, overall water splitting, CO_2_ reduction, nitrogen fixation, pollutant degradation, and microwave- or thermally driven systems, illustrating the versatility of field-assisted strategies and the mechanistic origins of their performance gains.
- Discuss advanced operando and field-resolved characterisation techniques that enable direct observation of field-catalyst interactions and carrier dynamics under reaction conditions.
- Identify key challenges and provide future perspectives.

In this review, external stimuli primarily refer to externally applied physical inputs, including electric fields, magnetic fields, thermal energy, and microwave irradiation. While photothermal catalysis also involves thermal effects, the heat originates from photon absorption rather than an independently applied external stimulus. We therefore discuss photothermal catalysis separately as a related phenomenon that provides useful insights into the role of thermal energy in photocatalytic systems.

Based on a critical assessment of the literature, we outline design principles and experimental strategies to resolve mechanistic ambiguities and support the rational development of next-generation field-assisted photocatalytic systems. The review proceeds by first establishing the fundamental physics of field-charge and field-lattice interactions (Sect.  2), followed by detailed analyses of individual stimuli, including representative materials, quantitative mechanisms, and design guidelines (Sects.  3, 4, 5, 6 and 7). We further discuss emerging multi-stimuli synergies and advanced operando characterisation techniques that reveal field-dependent charge, spin, and interfacial dynamics. The article concludes with future perspectives on key challenges and opportunities for translating external-stimuli-assisted photocatalysis into practical energy and environmental applications.

## Basic principles of photocatalysis

A typical photocatalytic reaction involves three fundamental steps [4, 24]:

(i) Photon absorption: excitation of electrons from the valence band (VB) to the conduction band (CB), leaving holes in the VB;
(ii) Charge separation and transport: migration of photogenerated charge carriers to the catalyst surface while minimising electron-hole recombination;
(iii) Surface redox reactions: transfer of electrons and holes to adsorbed reactants, driving the desired surface chemical conversions.

The overall efficiency of photocatalysis depends on the effectiveness of each step, which in turn is governed by the intrinsic properties of the catalyst, such as band structure, crystallinity, and surface states, as well as external reaction conditions including light intensity, temperature, and reactant concentration. An ideal photocatalytic system must therefore optimise all three processes simultaneously.

Although internal electric fields, such as those arising from band bending at semiconductor junctions or spontaneous polarisation in ferroelectric materials, can facilitate charge separation and interfacial reactions to some extent, these effects are typically limited in magnitude and spatial range. External stimuli, on the other hand, can provide additional or complementary driving forces that significantly influence carrier dynamics, interfacial charge transfer, and surface energetics, thereby offering new opportunities to enhance photocatalytic performance.

## Magnetic-field-assisted photocatalysis

Research on magnetic-field effects (MFEs) in photocatalysis has gained attention only recently, covering reactions such as nitrogen fixation, denitrification of contaminated water, CO_2_ conversion, and dye degradation [23, 25–29]. In these studies, external magnetic fields were applied during catalytic reaction. The reported enhancements are commonly attributed to improved adsorption of charged species, modulation of internal electric fields, and facilitated charge-carrier migration. A magnetic field interacts with charge carriers primarily through two mechanisms [23]:

(i) the Lorentz force, which alters the directions of photoexcited electrons and holes;
(ii) the Zeeman interaction, which separates spin-up and spin-down states and modifies spin-dependent reaction kinetics.

Both mechanisms influence the spatial and spin distributions of carriers, thereby affecting recombination dynamics and radical lifetimes in redox reactions. The net impact of a magnetic field on a semiconductor photocatalyst depends on factors such as carrier mobility, effective mass, magnetic susceptibility, and spin-orbit coupling. However, these effects are often intertwined, making it difficult to distinguish individual contributions. Moreover, many non-magnetic oxide semiconductors exhibit weak magnetic responses even under strong external fields, resulting in minimal performance enhancement. The underlying mechanisms of MFEs therefore remain insufficiently understood.

### Lorentz force and spin polarisation

**Fig. 1:**
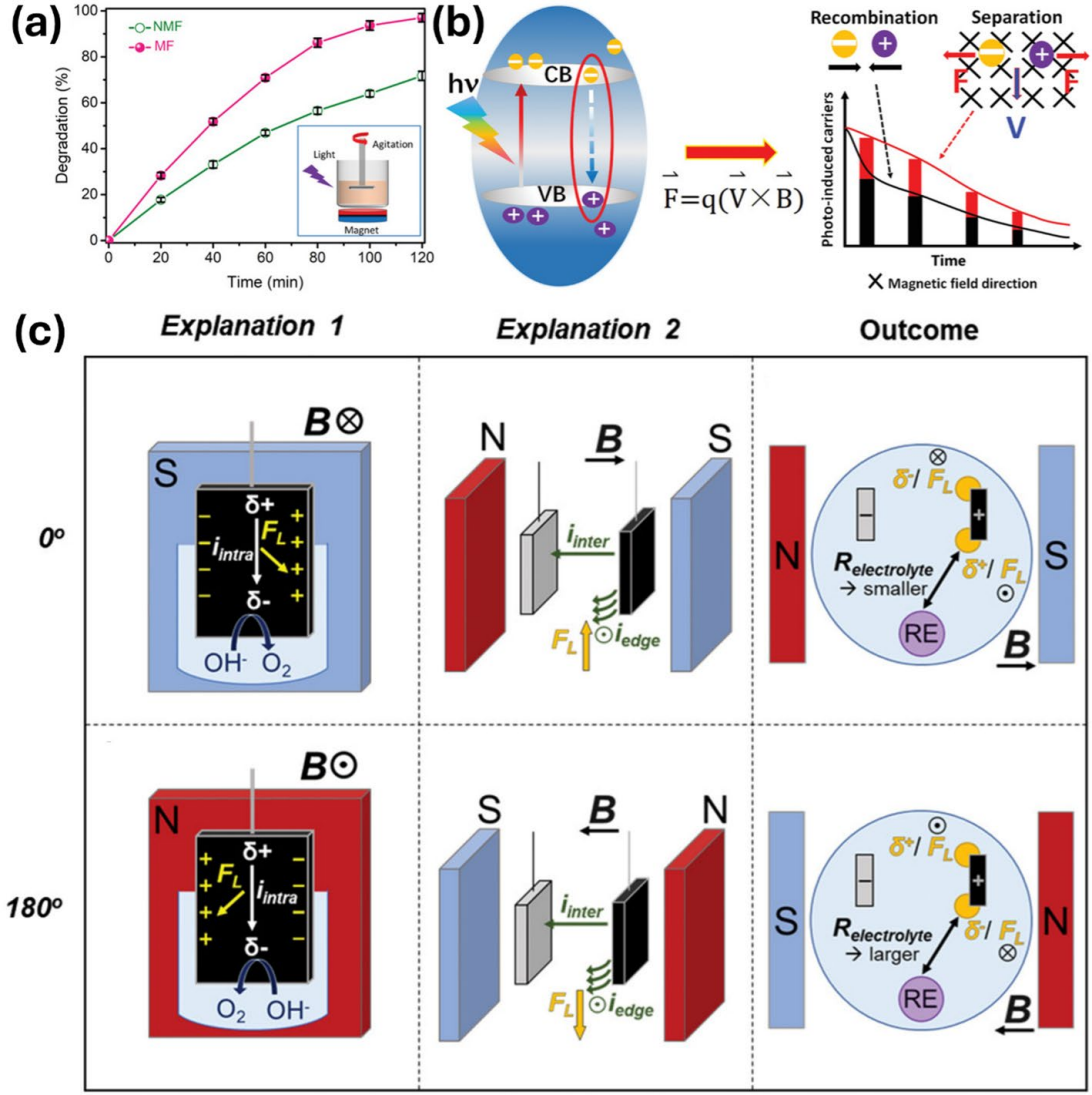
Magnetic field effects on photocatalytic and electrochemical systems. **a** Photocatalytic degradation of MO with no magnetic field (NMF) or with a magnetic field (MF) in the presence of TiO_2_ nanobelts under UV illumination. The inset is a schematic diagram of the magnetic field photocatalytic setup. **b** Schematic illustration of the proposed influence of the magnetic field on photoinduced charge carrier separation in the TiO_2_ nanobelts. Reproduced with permission from ref [30]. Copyright 2019 CC BY 4.0. **c** Schematic representations of magnetic field effects when the magnetic field is parallel or vertical to the electrodes. Reproduced with permission from ref [31]. Copyright 2025 CC BY 4.0

Lorentz-force and spin-polarisation effects are the most frequently reported contributors to MFEs. The Lorentz force acts on moving charges under an external magnetic field. Early studies showed that even weak magnetic fields can enhance photocatalytic degradation of organics on TiO_2_ [25, 28, 29]. For example, Gao et al. reported that a nearly 0.1 T field increased the methylene orange decomposition rate by 26%, attributed to Lorentz-force-induced separation of electrons and holes, effectively widening the space-charge region (Fig. 1a, b) [30]. In colloidal suspensions, moving charge carriers in a magnetic field induce microscopic electromotive forces that spatially separate reduction and oxidation sites on individual particles – analogous to the bipolar effect observed under electric fields.

The Lorentz force can also generate macroscopic fluid convection, known as magnetohydrodynamic (MHD) flow. MHD effects are particularly relevant in photoelectrochemical systems and depend strongly on electrode geometry, spacing, and reactor dimensions. Such convection influences mass-transport steps by promoting bubble detachment and reducing the diffusion-layer thickness at electrode surfaces (Fig. 1c) [31, 32].

It has been reported that synergistic Lorentz and spin-polarisation effects can substantially enhance overall photocatalytic water splitting (POWS) [33]. In a Au/Fe_3_O_4_/N-TiO_2_ composite, a modest external magnetic field (~ 180 mT) induced strong local flux from the Fe_3_O_4_ layer, yielding an exceptional solar-to-hydrogen (STH) efficiency of 11.9 ± 0.5%, surpassing the U.S. DOE 10% target for practical application. Time-resolved photoluminescence and density functional theory (DFT) analyses showed that the magnetic field prolonged exciton lifetimes by driving charge separation via Lorentz forces and suppressing recombination through spin-selective relaxation in spin-polarised defect states of N-TiO_2_ [33]. This coupled spin-charge modulation markedly improved carrier dynamics and redox kinetics, establishing magnetic-field-assisted photocatalysis as a promising non-contact approach for efficiency enhancement.

Similarly, in lanthanide-doped perovskite oxynitrides (Ln: BaTaO_2_N), our recent unpublished work has found that magnetic fields couple spin and lattice distortions to extend carrier lifetimes. The 4*f* electrons of Ln^3+^ ions introduce localised magnetic moments that interact with conduction-band states through *f*-*d* exchange, creating spin-polarised carriers. Under an external stimulus, ferromagnetic ordering stabilizes these interactions, enhancing spin polarisation, prolonging the charge carrier lifetime, and facilitating the oxygen vacancy formation. This spin-charge coupling increased photocatalytic efficiency by over 40% under simulated sunlight while maintaining structural stability.

The concept of spin-polarised surface chemistry has also expanded beyond magnetic oxides. In Au/CdS core-shell nanostructures, rotating magnetic fields induce electromotive currents in the metal core, shifting local potentials and doubling the H_2_-evolution rate [34]. This synergy between plasmonic hot-electron generation and magnetic spin polarisation highlights the emerging field of magneto-plasmonic photocatalysis. The OER further illustrates the role of spin. OER involves spin-dependent electron transfer: while OH^−^ (alkaline) and H_2_O (acidic) reactants are singlet (paired-spin) species, the product O_2_ exists mainly in a triplet ground state with two unpaired electrons. The required spin transition contributes to sluggish OER kinetics and high overpotentials. Under an external magnetic field, alignment of unpaired spins on catalyst sites can “pin” oxygen intermediates in favourable configurations, facilitating the formation of triplet O_2_ and improving reaction rates [35–37].

### Magnetoresistance effects

**Fig. 2:**
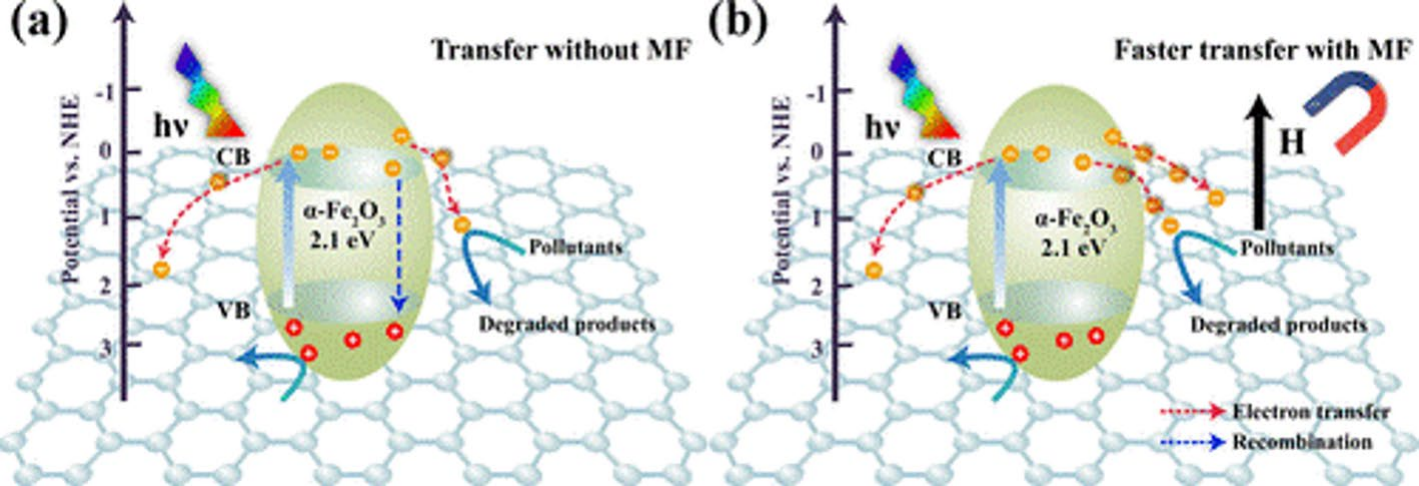
Schematic illustration of proposed photocatalytic mechanism in the α-Fe_2_O_3_/rGO composites without **a** and with **b** magnetic fields. Reproduced with permission from ref [26]. Copyright 2018 American Chemical Society

The magnetoresistance (MR) effect is often overlooked in photocatalysis (Fig. 2) [26, 32, 38, 39]. It describes how the electrical resistance of semiconductors changes in response to an applied magnetic field. A positive MR indicates increased resistance, while a negative MR implies decreased resistance. MR arises from several mechanisms: spin polarisation (negative MR via spin-aligned channels), Zeeman-induced spin-disorder scattering (positive MR), and Lorentz-force-driven carrier deflection (Hall effect). In photocatalysis, MR influences electron transport across catalyst interfaces and affects the diffusion of charged species in solution.

For instance, α-Fe_2_O_3_/reduced graphene oxide (rGO) composites exhibit negative MR under a 1000 Gauss field, leading to a ~ 30% increase in photocurrent density due to reduced interfacial scattering and improved electron injection from Fe_2_O_3_ to rGO [26]. A recent study found that MR can dominate the overall MFE, depending on the catalyst’s magnetic character and interfacial structure [31]. Ferromagnetic Ni-based electrodes show pronounced negative MR, which lowers charge-transfer resistance and decreases OER overpotentials. In contrast, weakly paramagnetic materials such as Pt exhibit small positive MR, where spin-disorder scattering reduces activity. The substrate also plays a role: ferromagnetic supports like Ni foam contribute additional negative MR, further improving electronic coupling between active species and supports.

Overall, MR provides a fundamental spin-electronic pathway by which magnetic fields can modulate catalytic activity. By tuning the magnetic nature of both catalysts and substrates, MR can be exploited to minimize interfacial resistance, enable spin-selective charge transport, and complement Lorentz and spin-polarization effects.

### Other magnetic field effects

Other magnetic effects may also influence photocatalysis. The Kelvin force acts on paramagnetic species in non-uniform magnetic fields, drawing them toward regions of higher field intensity and creating fluid convection. Given the typically low concentration of paramagnetic ions in photocatalytic systems, Kelvin force contributions are expected to be minor [40]. However, interactions with paramagnetic O_2_ (^3^O_2_) might hinder product diffusion near magnetic electrodes, which is yet to be systematically studied. The Maxwell stress describes surface deformation of polarisable species under a magnetic field [41]. It may distort the chemical environment near catalyst surfaces if paramagnetic species are present. Maxwell stress is negligible for rigid solid-state catalysts but may affect elastic electrolyte species.

In short summary, magnetic fields offer a non-contact and dynamically tuneable method to manipulate spin alignment and carrier migrations without altering catalyst composition. They are particularly attractive for spin-dependent reactions such as OER. However, the magnitude of magnetic effects depends strongly on material magnetic susceptibility, and many common semiconductors exhibit weak magnetic responses. In addition, disentangling Lorentz, Zeeman, and magnetoresistance contributions remains experimentally challenging.

## Electric-field-assisted photocatalysis

Among the various external stimuli, the electric field is conceptually the simplest and most versatile. It directly governs charge-carrier behaviour through electrostatic forces and can be introduced either by applying an external bias or by employing wireless bipolar electrochemical configurations. Systems utilising such approaches are generally classified as photoelectrochemical (PEC) systems, where the applied field facilitates charge separation, migration, and interfacial redox reactions.

### Conventional electric-field-assisted systems

In conventional photoelectrochemical setups, an applied bias facilitates charge extraction at photoelectrodes, but this configuration is limited to contact-based film geometries. Wireless electric-field assistance provides an alternative in which two driving electrodes generate a field across a suspension, inducing dipole formation within illuminated particles and accelerating redox reactions. Recent advances also extend this concept to wireless PEC architectures, where the solar absorber itself operates without external wiring. Moon et al. demonstrated a triple-junction photocathode protected by TiO_2_ that achieved 13% solar-to-hydrogen conversion efficiency, showing the potential of wireless configurations to minimise ohmic losses and simplify system architecture [42].

Electric fields can also tune ferroelectric semiconductors by aligning dipoles through poling, generating remanent polarisation that enhances internal band bending. Such control enables switchable interfacial energetics, as demonstrated in poled BiFeO_3_-BiVO_4_-CuInS_2_ heterojunctions, where reversing the poling direction reverses band alignment and activity [43]. Beyond charge separation, electric fields modulate adsorption behaviour. Ferroelectric-domain-dependent CO_2_ adsorption on BaTiO_3_ and polarization-selective dye adsorption on Bi_3_TiNbO_9_ illustrate how electric-field-induced surface potentials influence reactant binding [44, 45]. These effects offer opportunities for directional catalysis, patterning, and selective surface chemistry.

### Engineering of local electric field

Long carrier lifetimes are critical for efficient photocatalysis. While light absorption and carrier generation occur within femtoseconds, charge separation and migration take hundreds of femtoseconds to picoseconds, and interfacial charge transfer can extend to microseconds. Without sufficient lifetime, most photogenerated charge carriers recombine before reaching surface active sites. Strengthening local electric fields (LEFs), particularly through polar interfaces, has proven to be an effective strategy to enhance both bulk and surface charge separation and thereby extend carrier lifetimes.

It has been recently demonstrated a practical and versatile way to introduce strong interfacial LEFs by coupling photocatalysts with polar-faceted supports [46]. Polar surfaces such as MgO (111), CeO_2_ (100), and ZnO (0001) possess intrinsic surface dipoles that generate persistent electrostatic fields near the surface. When N-doped TiO_2_ was supported on MgO (111), time-resolved photoluminescence (TRPL) showed that the charge carrier lifetime increased from 2.56 ns to 5.76 ns. The resulting Au/N-TiO_2_/MgO (111) composite achieved a hydrogen evolution rate of approximately 11,092 µmol g^−1^h^− 1^ at 270 °C, with apparent quantum efficiencies (QEs) of 81.8% at 437 nm and 3.2% at 1000 nm. In contrast, non-polar MgO (100) and (110) surfaces produced no enhancement, confirming the key role of surface polarity. To exclude morphological effects, N-TiO_2_ particles of varying sizes were tested on identical MgO (111) supports. Smaller particles exhibited stronger LEF-induced enhancements, consistent with the field being localized at the interface. Similar behaviour was observed on other polar oxides such as CeO_2_(100) nanocubes and ZnO(0001) nanoplates, whereas non-polar counterparts showed negligible influence. The photocatalytic activity and charge carrier lifetime both scaled approximately linearly with calculated surface polarity, suggesting that LEF strength and thus photocatalytic performance can be rationally tuned through support selection.

**Fig. 3:**
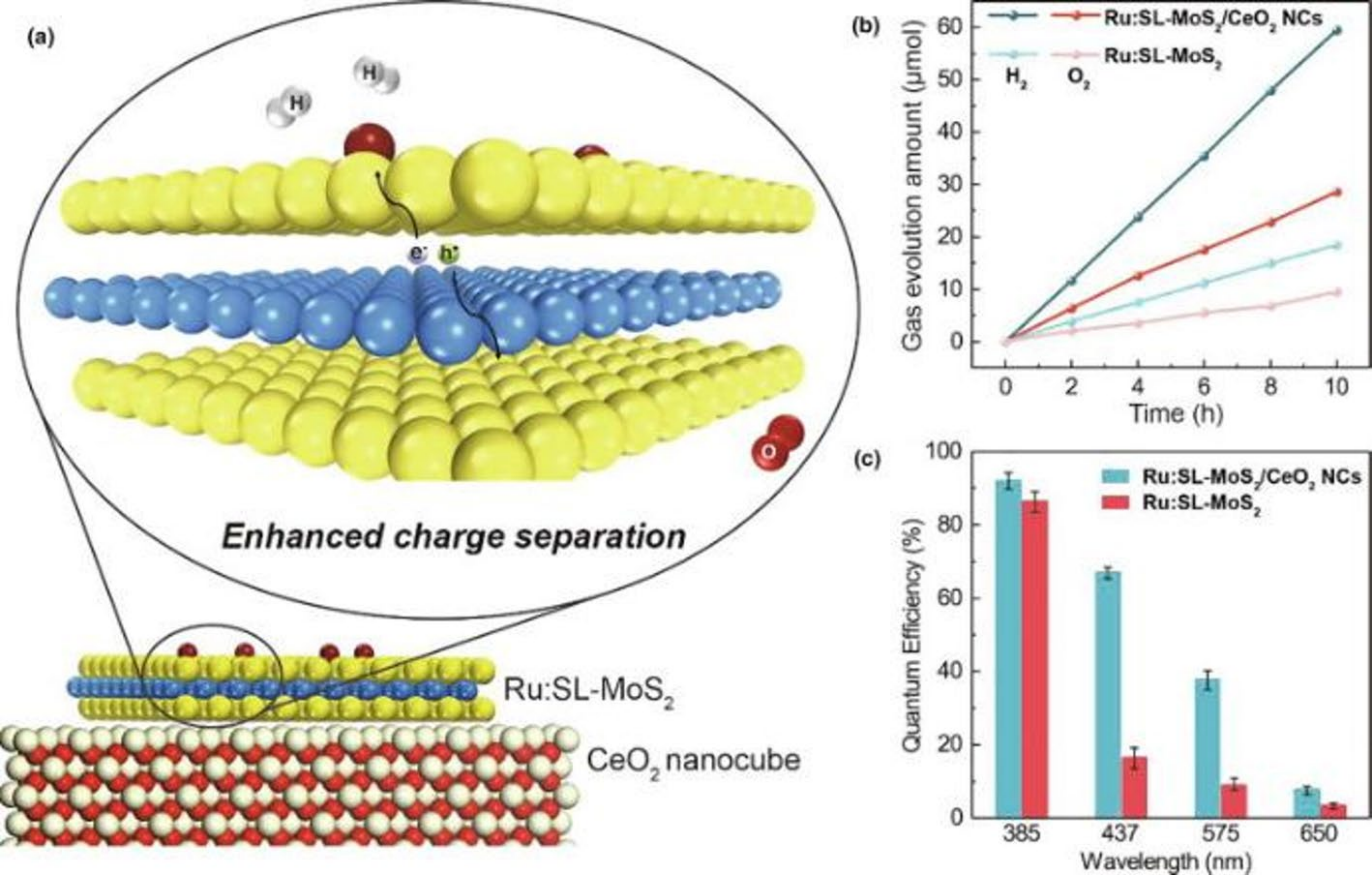
LEF effects on MoS_2_-based photocatalysts. **a** Schematic illustration of the charge separation process promoted by local polarisation introduced by polar-faceted oxide, resulting in enhanced photocatalytic water splitting performance. **b** Photocatalytic activity tests: stable stoichiometric decomposition of water at 270 °C over Ru: SL-MoS_2_ with and without polar CeO_2_ support. **c** QE of Ru: SL-MoS_2_ with and without polar CeO_2_. Reproduced with permission from ref [47]. Copyright 2020 Elsevier

The LEF strategy also extends beyond oxides. Two-dimensional metal chalcogenides such as single-layer MoS_2_ (SL-MoS_2_) are especially sensitive to interfacial polarization due to their structural flexibility, as shown in Fig. 3 [47]. In Ru-doped SL-MoS_2_ combined with polar supports, significant activity enhancements were achieved: Ru: SL-MoS_2_/CeO_2_(100) yielded a hydrogen evolution rate of around 2,977 µmol g^−1^h^− 1^, which is about four times higher than with non-polar supports. In all cases, charge carrier lifetimes and hydrogen evolution rates increased linearly with the total polarity of the support, confirming that localized interfacial polarization is the main driver for the observed performance gains.

Building on this concept, Li et al. recently demonstrated an electrolyte-assisted polarisation mechanism that enables efficient seawater splitting over facet-engineered N-TiO_2_ [48]. At 270 °C, ionic species in seawater (e.g., Na^+^ and Cl^−^) selectively adsorb onto photo-polarized (101) and (001) facets, generating local electric fields that prolong carrier lifetimes by up to fivefold. TRPL and in situ ambient-pressure XPS confirmed facet-dependent charge separation, while DFT calculations established linear correlations between photocatalytic activity, charge carrier lifetime, and the calculated electrolyte-induced polarization energy. This cooperative effect produced an overall energy conversion efficiency of 15.9 ± 0.4% and a STH efficiency up to 20.2 ± 0.5% under concentrated solar illumination, corresponding to hydrogen evolution rates comparable to laboratory-scale electrolysers. These results demonstrate that ionic adsorption at elevated temperatures can enhance local electric fields, providing a simple and scalable route to enhance charge separation and extend carrier lifetimes in saline and real seawater environments.

In conclusion, electric fields provide direct control over band bending, carrier separation, and interfacial energetics, enabling reversible and switchable photocatalytic behaviour. Ferroelectric materials further allow remanent polarisation without continuous energy input. Nevertheless, field screening in liquid environments, charge accumulation effects, and long-term material stability under repeated poling remain important challenges. Scalable and energy-efficient field application also requires careful reactor design.

## Thermal-assisted photocatalysis

Since most photocatalytic systems operate at room temperature [4, 24, 49], here we regard heat as an external stimulus. Emerging reports have demonstrated that elevated reaction temperatures can enhance photocatalytic performance both thermodynamically and kinetically [50–52]. For example, direct thermolysis of water requires impractically high temperatures (> 1000 °C), yet modest heating combined with photoexcitation offers a practical route to boost photocatalytic performance [46, 48, 53].

Recent studies have demonstrated the potential of temperature-assisted photocatalysis. Han and Hu showed a visible light QE of 65.7% at 280 °C in a sacrificial hydrogen evolution system; however, methanol thermolysis contributes to H_2_ production and produces CO_2_, which limits practical application [54]. Tian et al. reported an around 9-time enhancement in water splitting over black phosphorus nanosheets at 80 °C, achieving a QE of 42.55% at 430 nm without sacrificial reagents [55]. However, oxygen was not produced stoichiometrically in that work, suggesting photocorrosion of the phosphide catalyst.

**Fig. 4:**
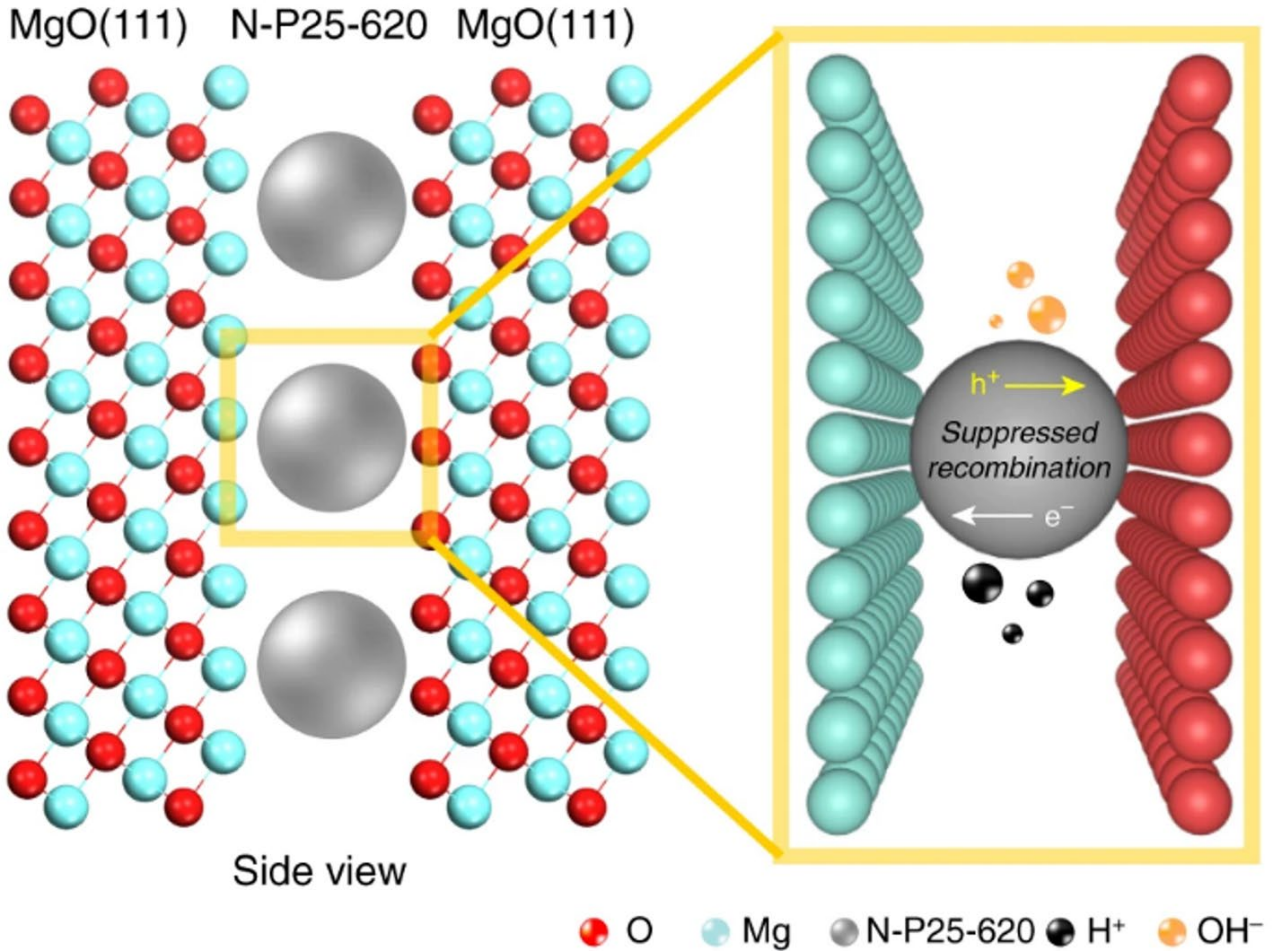
Schematic illustration of the local electric field of polar MgO (111) nanocrystals with negative and positive ion-terminated surfaces can assist the photocatalytic water splitting to H_2_/O_2_ via H^+^ and OH^−^ surrounding the N-doped TiO_2_ catalyst particle. Reproduced with permission from ref [46]. Copyright 2019 CC BY 4.0

**Fig. 5:**
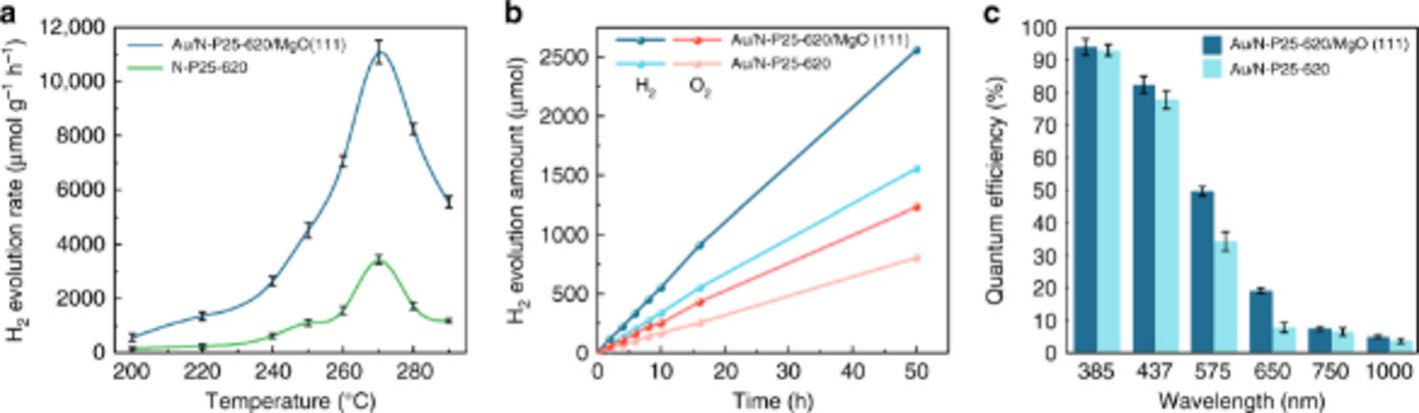
Photocatalytic water-splitting reaction activity tests. **a** Photocatalytic activities of N-TiO_2_ catalysts at different temperatures. **b** Stable stoichiometric decomposition of water to 2:1 H_2_/O_2_ with no sacrificial reagent over Au/N-P25-620 with and without MgO (111) at a constant rate for 50 h. **c** QE of Au/N-P25-620 with and without MgO (111) by using incident wavelengths of 385, 437, 575, 620, 750 and 1000 nm, respectively. Reproduced with permission from ref [46]. Copyright 2019 CC BY 4.0

Building on these previous reports, Li et al. developed several thermal-assisted POWS systems (Fig. 4) [33, 46–48, 53, 56, 57]. Exceptional efficiencies were achieved, with QEs of 81.8% at 437 nm and 3.2% at 1000 nm at 270 °C on TiO_2_-based catalysts. Electron paramagnetic resonance (EPR) revealed that surface oxygen vacancies (V_O_) in N-doped TiO_2_, which readily passivated by air at room temperature, are rapidly regenerated at elevated temperature in nitrogen, liquid water, or water vapour. Thus, high temperature simultaneously sustains active surface defects and accelerates the reactions they mediate. Temperature also promotes water ionic dissociation, with the equilibrium concentrations of H^+^ and OH^−^ increasing sharply and peaking near 250–270 °C, orders of magnitude higher than that at ambient conditions (Fig. 5a). These higher ionic concentrations improve interfacial redox kinetics and influence charge carrier dynamics, leading to enhanced photocatalytic performance. TRPL further revealed that while carrier lifetimes show little change with temperature in air, they increase markedly in aqueous environments. This behaviour indicates that adsorbed H^+^ and OH^−^ species induce LEFs at the semiconductor surface, which attract counter charges, suppress recombination, and prolong carrier lifetimes (Fig. 5b, c). Heat therefore acts not only to accelerate reaction kinetics but also to strengthen interfacial polarisation, making it an especially effective external stimulus for photocatalysis.

To fully utilise both photochemical and thermal energy from sunlight, Li et al. established an integrated photocatalytic-photothermal (PC-PT) conversion system. In this unpublished work, elevated temperature also activates the lattice oxygen mediated (LOM) pathway for oxygen evolution, while defect engineering minimizes radiative recombination and enhances non-radiative (thermal) relaxation. The photothermal energy stored in superheated water can be reused for additional hydrogen production or electricity generation, forming a closed-loop hydrogen-plus-heat system. From a practical perspective, future systems could be even more efficient when heat is supplied by concentrated solar light rather than external electrical heaters. Concentrated light provides both illumination and heat, as demonstrated using a light-concentrating furnace capable of sustaining stable POWS operation at 270 °C solely with solar input [46]. Moreover, superheated water carries substantial thermal energy that could be harnessed downstream, for instance, via steam-turbine energy generation.

As mentioned earlier, we also showed that coupling magnetic fields with elevated temperatures can further enhance photocatalytic performance. Mechanistic analysis combining TRPL and DFT revealed that high temperature facilitates oxygen-vacancy regeneration and improves charge mobility, while an external magnetic field drives Lorentz-force-based charge separation and suppresses recombination through spin-polarized states in N-TiO_2_ [33]. The synergistic effects of thermal, magnetic, and spin polarisation significantly extend exciton lifetimes and offer a non-contact route to boost photocatalytic efficiency.

Overall, these thermal, magnetic, and local-field effects become prominent only at elevated temperatures, where sluggish oxygen evolution kinetics can be overcome by enhanced oxygen vacancy migration and lattice oxygen participation. Heat not only accelerates intrinsic reaction kinetics but also activates defect dynamics and interfacial polarisation processes that improve charge separation. Altogether, thermal-assisted photocatalysis provides a practical and powerful route to significantly increase quantum efficiencies and reaction rates, paving the way for high-performance, self-sustaining solar hydrogen production.

Beyond homogeneous heating, temperature gradients themselves can be exploited to drive charge separation through pyroelectric and thermoelectric effects. In pyroelectric materials, temporal temperature fluctuations induce changes in polarisation, generating alternating surface charges that push electrons and holes in opposite directions during heating-cooling cycles. This dynamic polarisation acts as a periodic “charge pump” [58, 59]. Temperature gradients also introduce opportunities in magnetic semiconductors, where thermo-spin coupling adds a spin-dependent dimension to thermal effects. In ferromagnetic LaFeO_3_, for instance, a ΔT of only ~ 10 K under visible-light irradiation produces measurable spin-polarised photocurrents [60]. Although still an emerging area, such thermo-spin and magnetothermal interactions may offer new strategies for enhancing photocatalytic reactivity under thermal gradients.

Although photothermal catalysis is conceptually distinct from thermal-assisted photocatalysis because the thermal energy originates from photon absorption, recent photothermal studies provide valuable mechanistic insight into the role of heat in catalytic processes. In the photothermal dry reforming of methane over Ce-promoted Ni/NiO heterostructures, light-driven heating significantly reduced activation barriers and enhanced CH_4_ conversion by promoting oxygen vacancy formation and improving charge transfer between Ni and CeO_2_ domains [61]. The synergistic interaction between photothermal excitation and defect-mediated catalytic sites enabled sustained activity at relatively moderate temperatures compared to purely thermal processes. Similarly, in Ru/CeAlO_x_ systems for CO_2_ hydrogenation, the coexistence of atomically dispersed Ru single atoms and Ru nanoparticles created complementary active sites, where light-induced local heating and charge carrier activation enhanced H_2_ dissociation and CO_2_ adsorption [62]. Under full-spectrum irradiation, the catalyst exhibited significantly higher CH_4_ production rates compared to dark conditions, confirming that photon-driven thermal effects and electronic excitation can operate cooperatively. These studies demonstrate that under high-intensity illumination, photothermal contributions become non-negligible and can synergistically couple with catalytic defect chemistry and charge-transfer processes, blurring the boundary between purely photocatalytic and thermal catalytic regimes.

To briefly summarise, elevated temperatures enhance reaction kinetics, defect mobility, ionic dissociation, and lattice oxygen participation, often leading to dramatic improvements in quantum efficiency. Thermal assistance is also readily compatible with concentrated solar systems. However, high-temperature operation may accelerate material degradation, induce sintering, and complicate mechanistic separation between thermal and photochemical contributions. Precise thermal management is therefore critical.

## Microwave-assisted photocatalysis

Microwave radiation (300 MHz − 300 GHz) interacts with materials through the oscillation of dipoles and conduction electrons. Unlike conventional heating, microwaves deposit energy volumetrically within the material, resulting in rapid and uniform temperature increases. In photocatalytic systems, both thermal and non-thermal effects contribute to activity enhancement [63–69]. The thermal component arises from uniform heating, which increases charge carrier density and reaction rates. Non-thermal effects originate from field-induced polarisation of defects and ions, which can alter electronic structures and generate localised “hot spots” that serve as reaction active sites. The existence of genuine non-thermal microwave effects remains controversial, as many reported enhancements may alternatively arise from localized heating, dielectric heterogeneity, or hot-spot formation rather than direct field-induced changes in catalytic energetics.

Microwave irradiation also acts as a versatile tool for defect engineering. It can generate surface oxygen vacancies and cation defects that introduce mid-gap states, extending optical absorption into the visible range. For instance, Horikoshi et al. reported the appearance of a new absorption band around 450 nm in TiO_2_ after only 10 min of microwave exposure, attributed to vacancy formation [70]. Transient absorption spectroscopy further indicated slower carrier recombination, confirming that these defects serve as shallow electron traps. Controlled microwave pretreatment thus offers a physical means of tuning defect structures and band gaps without chemical doping.

When microwaves and light are applied simultaneously, the oscillating electromagnetic field can modulate carrier mobility and interfacial potential, promoting dynamic charge separation. In situ EPR measurements have shown that microwave exposure increases the intensity of DMPO-•OH signals in TiO_2_/H_2_O systems, indicating enhanced radical generation [71]. Complementary Raman and infrared analyses revealed changes in the adsorption behaviour of organic molecules, consistent with field-driven polarisation of surface functional groups. Mechanistically, the alternating field periodically tilts the semiconductor band edges, leading to a time-dependent separation of electrons and holes analogous to alternate-current poling in ferroelectrics. In principle, the synchrony between the oscillating field and photon excitation frequency can be tuned for resonance enhancement of photocatalytic activity [72].

A practical implementation of this concept is the microwave electrodeless lamp reactor, where microwave energy excites a plasma lamp that emits UV-visible light while simultaneously exposing the catalyst to the microwave field [73]. This dual action accelerates photocatalytic oxidation reactions; complete degradation of organic pollutants such as pentachlorophenol or atrazine has been achieved within minutes. The observed synergy originates from rapid interfacial heating, increased defect concentrations, and enhanced radical mobility. For solid-gas reactions such as CO_2_ reduction, microwave-transparent reactors with dielectric windows allow selective heating of the catalyst while maintaining lower reactant temperatures, which improves product selectivity toward CO or CH_4_ [74].

For safe and efficient operation, uniform field distribution is crucial to avoid localised overheating or “hot spots,” which can cause nanoparticle sintering. Reactor designs incorporating mode stirrers and dielectric matching layers help to homogenise field intensity. Energy-efficiency assessments indicate that microwave-assisted photocatalysis can reduce overall energy consumption by up to 60% compared with conventional thermal processes when properly optimized [75–77]. Although several studies report improved reaction rates and reduced energy consumption compared with conventional thermal processes, quantitative comparison of microwave-assisted photocatalysis remains challenging. Differences in reactor geometry, dielectric properties of reaction media, microwave coupling efficiency, and power-normalisation methods make direct benchmarking difficult. At present, there is no universally adopted standard for evaluating energy efficiency in microwave-assisted photocatalytic systems. Future studies would benefit from standardised reporting of absorbed power density, thermal profiles, and photon-to-product energy balances to enable more reliable cross-study comparisons.

In summary, microwave irradiation enables rapid volumetric heating and defect engineering while potentially generating non-thermal polarisation effects. It can significantly shorten reaction times and enhance radical formation. However, quantitative benchmarking remains difficult due to variations in absorbed power density and reactor geometry. Non-uniform field distribution and localised overheating may also limit scalability and long-term stability.

## Advanced characterisation techniques

Understanding the complex interplay between external stimuli and photocatalytic processes requires advanced ex situ, in situ, and operando characterisation techniques capable of resolving field-dependent charge, spin, lattice, and interfacial dynamics. Because external stimuli introduce additional degrees of freedom, such as polarisation, spin alignment, defect migration, and local heating, conventional steady-state measurements are often insufficient to capture the true mechanistic picture.

Time-resolved spectroscopic methods are particularly powerful for probing dynamic carrier behaviour. TRPL enables direct quantification of carrier lifetimes and can reveal field-induced prolongation of charge separation through enhanced polarization or spin alignment [48]. Transient absorption spectroscopy (TAS), especially when combined with synchronous field modulation, provides insight into ultrafast carrier trapping, recombination kinetics, and polarisation processes [78–80]. In situ electron paramagnetic resonance (EPR) is uniquely suited to detect field-induced radical species and spin-polarised intermediates, offering direct evidence for magneto-spin effects and defect formation under operational conditions. As thermal-assisted photocatalysis attracts increasing attention, high-temperature operando TRPL, TAS, and EPR measurements are increasingly important for capturing thermally activated defect migration, lattice oxygen participation, and coupled spin-charge dynamics that cannot be observed under ambient conditions.

Operando X-ray and vibrational techniques provide complementary structural and chemical information. Synchrotron-based X-ray absorption spectroscopy (XAS) and X-ray photoelectron spectroscopy (XPS) can track reversible changes in oxidation states and band alignment under applied fields, such as Ti(IV)/Ti(III) transitions associated with oxygen vacancy formation. Operando Raman and Fourier-transform infrared (FTIR) spectroscopy allow monitoring of strain-induced lattice distortions, surface hydroxyl dynamics, and field-stabilized adsorbate configurations. These methods are particularly valuable for distinguishing genuine field-induced catalytic pathways from purely thermal or mass-transport effects.

Beyond experimental tools, integrated multiscale modelling plays a critical role in interpreting field-assisted phenomena. DFT calculations incorporating explicit external-field terms can predict changes in band structure, adsorption energies, and spin polarisation. Molecular dynamics simulations provide insight into temperature-driven defect mobility and interfacial polarisation, while reactor-level multiphysics simulations couple electromagnetic fields, heat transfer, mass transport, and reaction kinetics. Together, these theoretical approaches bridge atomistic mechanisms and macroscopic performance, enabling rational design of field-responsive catalytic systems.

## Future directions and perspectives

### Multi-stimuli and coupled-field photocatalysis

Although individual external stimuli, such as heat, electric fields, magnetic fields, or microwaves, each offer distinct benefits for enhancing photocatalysis, future catalytic systems are expected to operate under multiple simultaneous fields. This shift reflects not only practical reactor environments but also the growing recognition that these stimuli engage different physical degrees of freedom in the catalyst. Electric fields regulate band bending and charge migration; magnetic fields tune spin alignment and influence charge carrier dynamics; thermal fields accelerate reaction kinetics and defect mobility. When combined, their effects are presumably not linearly additive. Instead, non-linear synergies may arise because one field alters the material’s polarisation, spin texture, or electronic response to another.

Ferroelectric materials are a natural example of such coupling. As materials that are inherently both piezoelectric and pyroelectric, ferroelectrics exhibit spontaneous polarisation that can be modulated by stress or temperature gradients, thereby amplifying internal electric fields and influencing catalytic activity. Similarly, applying a magnetic field to piezoelectric or ferroelectric catalysts can steer spin-polarised carriers already driven by internal electric fields, producing cooperative enhancement of charge separation and spin ordering. Experimentally, the most mature demonstration of multi-field synergy is probably found in ferroelectric-piezoelectric coupling. In BiFeO_3_/TiO_2_ core-shell nanocomposites, electric poling aligns ferroelectric domains in BiFeO_3_, while ultrasonic vibration generates oscillating piezo-potentials. Together, they create significantly larger band bending at the interface, accelerating directional charge transfer. Under light and ultrasound, poled samples show over 235% higher organic dye degradation rates than unpoled ones [81].

Magnetic fields introduce yet another layer of control. As discussed earlier, they influence photocatalysis through modifications of charge carrier dynamics, alterations in spin alignments, and micro-convection near electrode surfaces. In magneto-photo-electrocatalytic systems, it is recently demonstrated that the orientation between magnetic field and current vector is critical: aligning the field parallel to current increases oxygen-evolution activity by over 60%, whereas a perpendicular configuration reduces performance due to lateral Lorentz drift [31, 32]. Incorporating magnetic nanoparticles within catalyst matrices further creates localized magnetic flux “hot spots,” which could be strategically engineered for future designs [33].

Light itself can couple with other fields in surprisingly rich ways. Because illumination provides an oscillating electromagnetic field, synchronising it with externally applied electric or microwave fields can lead to resonant phenomena. In Au/BaTiO_3_ plasmonic heterostructures, for instance, plasmon excitation under vibration amplifies local piezo-potentials, producing substantial electric fields, which dramatically accelerate hot-electron injection and surface reaction rates [82].

Given these opportunities, several principles will guide the design of next-generation multi-stimuli systems:

- Field compatibility: External stimuli must have spatial and temporal profiles that reinforce each other. Misalignment (e.g., opposite field polarity) can diminish or cancel beneficial effects.
- Material responsiveness: Catalysts with multi-functional characteristics, such as ferroelectricity, piezoelectricity, magnetism, semi-conductivity, offer stronger, intrinsic coupling between stimuli.
- Reactor integration: Future reactors must enable simultaneous control of illumination, magnetic flux, thermal gradients, and electric bias, ensuring reproducibility and quantitative correlation.

It should be noted that the simultaneous presence of multiple external stimuli does not necessarily imply true coupled-field synergy. In many reported systems, different stimuli contribute independently to photocatalytic enhancement. Strictly speaking, synergistic coupling should be reserved for cases where the combined effect exceeds the sum of the individual contributions. While several promising examples have emerged, experimentally validated demonstrations of such nonlinear coupling remain relatively scarce.

A comparison of the major external stimuli highlights their distinct yet complementary mechanistic roles. Electric fields directly modulate band bending, carrier separation, and interfacial energetics, enabling reversible and switchable control but facing challenges related to charge screening and stability in liquid environments. Magnetic fields primarily influence spin alignment, charge carrier migration, and interfacial charge transport, making them particularly relevant for spin-dependent reactions such as OER, although their effectiveness depends strongly on the magnetic properties of the catalyst. Thermal assistance enhances intrinsic reaction kinetics, defect mobility, ionic dissociation, and lattice oxygen participation, often leading to substantial improvements in quantum efficiency, yet high-temperature operation requires careful management of durability and energy balance. Microwave irradiation combines rapid volumetric heating with potential non-thermal polarisation effects and defect engineering capabilities, but quantitative benchmarking and scale-up remain challenging due to reactor-dependent energy coupling and field distribution. Rather than competing strategies, these external stimuli offer complementary mechanistic levers that may be most powerful when rationally integrated in coupled-field systems.

To facilitate comparison across different external-stimuli-assisted photocatalytic systems, Table 1 summarizes representative examples using consistent descriptors, including stimulus type and intensity, catalyst composition, reaction type, benchmark performance metrics, and the dominant mechanistic role. Because experimental conditions and reporting formats vary widely across studies, the table is intended as a curated comparison rather than an exhaustive or strictly normalized dataset. The selected examples were chosen to represent the major stimulus classes discussed in this review and to highlight general mechanistic trends rather than direct one-to-one performance ranking.

**Table 1:** Representative state-of-the-art external-stimuli-assisted photocatalytic systems

External stimulus	Catalyst system	Reaction	Key performance	Dominant mechanism	Refs.
Magnetic field	Au/Fe_3_O_4_/N-TiO_2_	Overall water splitting	STH 11.9 ± 0.5%	Lorentz-force separation; spin-polarised defect states	[33]
	α-Fe_2_O_3_/rGO	Photocurrent enhancement	~ 30% increase under 1000 G	Magnetoresistance; improved interfacial transport	[26]
Electric field (wireless PEC)	Triple-junction cell/TiO_2_	Water splitting	13% STH	Bias-driven charge extraction	[42]
Local electric field	N-TiO_2_/MgO (111)	POWS	QE 81.8% (437 nm), 3.2% (1000 nm)	Interfacial polarisation; carrier lifetime extension	[46]
	N-TiO_2_	Seawater splitting	STH 20.2 ± 0.5%	Electrolyte-induced polarisation	[48]
Thermally assisted	Au/N-TiO_2_	POWS	QE 81.8%	Defect regeneration; ionic dissociation	[46]
	Ni/NiO-CeO_2_	CH_4_ dry reforming	Enhanced CH_4_ conversion	Vacancy formation; interfacial charge transfer	[61]
	Ru/CeAlO_x_	CO_2_ hydrogenation	High CH_4_ selectivity (~ 100%)	Synergy of single atoms and NPs; light-induced heating	[62]
Microwave	TiO_2_	Pollutant degradation	Accelerated radical generation	Dielectric heating; defect engineering	[70]
	Zn ferrite/TiO_2_	Tetracycline degradation	Complete degradation within minutes	Rapid heating; enhanced radical mobility	[73]

### Challenges

Despite significant progress, several bottlenecks remain before multi-field photocatalysis reaches technological maturity.

Quantifying local field effects remains a major challenge. Macroscopic parameters such as applied voltage, magnetic flux density, microwave power, or reactor temperature rarely reflect the actual local fields experienced by catalyst particles. Dielectric screening, particle morphology, interfacial charge accumulation, and local heterogeneity can generate substantial differences between nominal and effective field strengths. Developing field-resolved operando techniques capable of probing local electric, magnetic, and thermal environments will be essential for establishing quantitative structure–performance relationships.

Decoupling multiple mechanisms is equally difficult. External stimuli often introduce several concurrent effects that operate over different temporal and spatial scales. For example, magnetic fields may simultaneously influence carrier transport, spin alignment, and mass transport, while microwave irradiation can generate both dielectric heating and field-induced polarization effects. Similarly, elevated temperatures affect reaction kinetics, defect mobility, ionic dissociation, and carrier dynamics simultaneously. Combining well-defined model systems with advanced operando characterization and multiphysics simulations will be crucial for distinguishing individual contributions and identifying the dominant mechanisms.

Material durability and long-term stability must also be carefully considered. Repeated electrical and mechanical cycling can induce domain fatigue, microcrack formation, or phase transitions in ferroelectric and piezoelectric materials. Elevated temperatures may accelerate catalyst sintering, defect annihilation, or structural degradation, while prolonged microwave exposure can alter catalyst morphology and dielectric properties. Future development of robust field-responsive materials will therefore require systematic lifetime testing under realistic operating conditions.

Energy efficiency and scale-up represent perhaps the most important challenge for practical implementation. For thermal-assisted systems, particularly those operating at elevated temperatures, careful evaluation of net energy balance is essential. Although substantial increases in quantum efficiency can be achieved, the energy required to generate and maintain external stimuli must be justified by the additional chemical energy produced. Future studies should report system-level energy efficiencies that incorporate heating, field generation, and auxiliary energy consumption in addition to photocatalytic performance metrics. Strategies such as concentrated solar heating, waste-heat utilisation, efficient electromagnetic-field generation, and heat-recovery systems may help improve overall sustainability. Ultimately, the energy cost of generating external stimuli must remain lower than the additional chemical energy gained if these approaches are to become technologically viable.

### Future perspectives

The next generation of photocatalysts is in urgent demand. Multifunctional perovskites, 2D ferroelectrics, magnetic semiconductors, and flexible polymers will enable simultaneous photo-, piezo-, pyro-, and magneto-responses. A major frontier is the coherent control of spin-charge-lattice interactions. Strategically combining electric and magnetic fields could engineer spin-selective pathways and suppress non-radiative recombination, potentially approaching unity quantum efficiency.

Field-assisted photocatalysis also offers strong synergies with renewable-energy technologies. Solar concentrators can provide combined light and heat; waste heat or mechanical vibration from industry can serve as “free” external stimuli; and geomagnetic or engineered magnetic environments may enhance plasmonic or spin-selective reactions. Applications may extend to magneto-plasmonic CO_2_ reduction, ultrasonic water splitting, microwave-assisted pollution remediation, and so on.

Recent advances in bifunctional photocatalysis further demonstrate the potential of simultaneously utilizing photogenerated electrons and holes in complementary reaction pathways. For example, coupled solar-fuel production and selective organic synthesis can significantly improve overall energy utilization and product value. Such “dual-benefit” strategies may provide inspiration for future external-stimuli-assisted systems, where external fields could be employed to selectively direct charge carriers toward different reaction channels [83, 84]. The ultimate vision is field-synergistic catalysis, a regime where optical, mechanical, electric, magnetic, and thermal stimuli operate coherently to guide charge carriers along designed pathways. Achieving this will require interdisciplinary collaboration but promises a transformative approach to solar chemical conversion.

A broader question is whether the incorporation of increasingly intense external stimuli may gradually shift photocatalysis toward conventional catalytic operation. While external fields often improve performance, they also introduce additional energy inputs that may reduce the traditional advantage of photocatalysis under mild conditions. Future development should therefore focus not only on maximizing activity but also on maintaining favourable system-level sustainability and energy efficiency.

## Conclusions

To provide a clearer overview of the current state of the field, Table 1 summarises representative external-stimuli-assisted photocatalytic systems. The selected examples span magnetic, electric, thermal/photothermal, and microwave stimuli, illustrating how different external inputs regulate charge separation, spin dynamics, defect chemistry, and interfacial energetics. This comparative summary highlights both the diversity of material platforms and the common mechanistic themes that underpin performance enhancement, thereby offering a structured reference point for evaluating progress and identifying future opportunities.

**Fig. 6:**
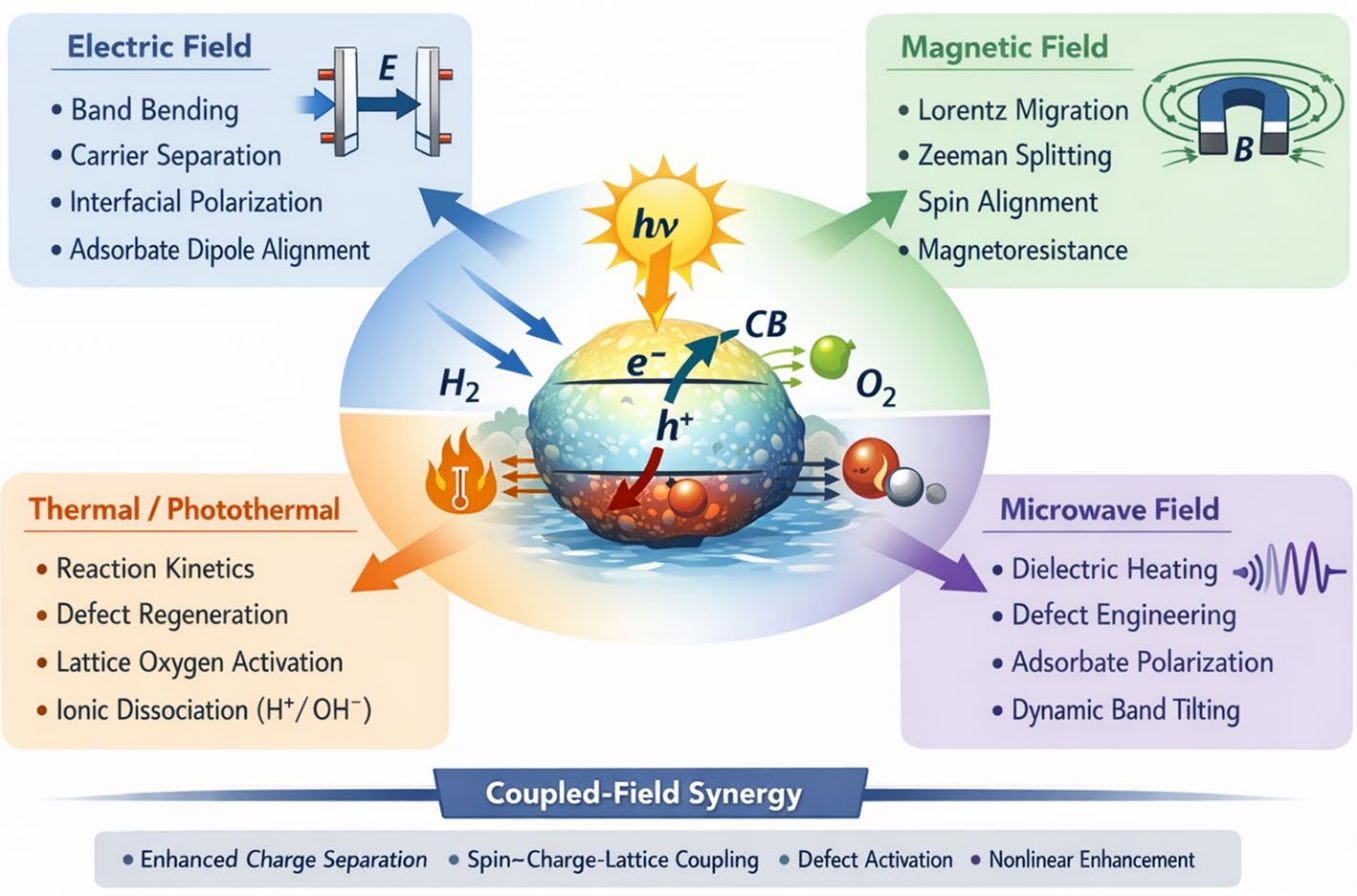
Conceptual overview of external-stimuli-assisted photocatalysis. Electric, magnetic, thermal/photothermal, and microwave stimuli interact with semiconductor photocatalysts through distinct physical mechanisms. These stimuli regulate charge, spin, lattice, and interfacial processes, and may generate nonlinear enhancements when combined in coupled-field systems

Clearly, external stimuli assisted heterogeneous photocatalysis represents a transformative direction of traditional photocatalysis, shifting the field from static materials engineering toward dynamic, field-responsive catalytic systems. By integrating electric, magnetic, thermal, microwave, and coupled-field stimuli, researchers have begun to access new regimes of charge separation, spin manipulation, interfacial polarisation, and reaction-pathway control that are unattainable through classical semiconductor design alone. External stimuli offer flexible, in-situ methods to modulate photocatalytic reactivity without altering catalyst chemistry (Fig. 6). Together, these stimuli unlock new kinetic and thermodynamic pathways for solar-driven chemical conversions, extending the operational landscape far beyond what intrinsic material properties alone can provide. Especially, photocatalysis at elevated temperatures has been proved an effective strategy for achieving significantly better solar energy conversion efficiencies.

Major challenges remain external-stimuli-assisted photocatalysis can be widely adopted. Quantifying the true local field strength within suspensions or porous catalysts remains difficult due to dielectric screening and interface complexity. Multi-stimuli systems introduce intertwined effects that require sophisticated experimental decoupling. Long-term material durability under repeated field cycling must be addressed, and reactor energy efficiency must be carefully evaluated to ensure a positive overall energy balance. Looking forward, dynamic and field-responsive catalysts, which are capable of adaptively tuning their electronic structure, polarisation, and spin texture in real time, offer a pathway toward overcoming intrinsic recombination limits. Meanwhile, data-driven discovery and operando spectroscopies capable of high-temperature measurements will accelerate rational catalyst design.

In summary, external-stimuli-assisted photocatalysis provides a versatile platform to modulate light, fields, and materials into a unified catalytic strategy. As mechanistic understanding deepens and reactor technologies mature, external-stimuli-assisted photocatalysis may offer a practical route to adaptive, high-efficiency solar chemical conversion. The convergence of controllable external stimuli with semiconductor photophysics not only expands the toolbox for photocatalyst optimisation but also opens new scientific directions in coherent field-synergistic catalysis, with promising implications for sustainable energy and environmental technologies.

## Data Availability

No datasets were generated or analysed during the current study.
